# Combining micro-plasma radio-frequency with hypofractionated electron-beam radiation as a novel treatment of keloids: A case series

**DOI:** 10.1097/MD.0000000000018094

**Published:** 2019-11-27

**Authors:** Wenchao Zhang, Zhifei Liu, Lin Zhu, Ang Zeng, Wenyun Ting, Xiaojun Wang, Nanze Yu, Guangpeng Xia

**Affiliations:** Plastic and Reconstructive Surgery, Department of Plastic Surgery, Peking Union Medical College Hospital, Beijing, China.

**Keywords:** electron-beam radiation, keloids, micro-plasma, plasma skin regeneration, radio frequency

## Abstract

**Rationale:**

Micro-plasma radio-frequency (MPR) technology has been demonstrated a safe and effective treatment for kinds of scars, but there is no report about the application of the MPR on keloids. In this investigation, we creatively use MPR technology combining with hypofractionated electron-beam radiation to cure keloids.

**Patient concerns and Diagnoses:**

From February 2013 to December 2016, 22 Asian patients (16 male, 6 female, age 19–46 years, mean age 28.14 ± 7.31 years) with keloids over half a year were enrolled in this study.

**Interventions and Outcomes:**

All patients received a single MPR technology treatment by roller tip at 80–100 watt, and then hypofractionated electron-beam radiation of 6 MeV were performed twice, within 24 hours and one week after the operation with 9 Gy per time. Improvement were determined by the Vancouver Scar Scales (VSS) according to digital photographs. The results show that the volume of keloids reduced significantly among most patients. Only 3 patients encountered with mild to moderate hyperpigmentation, and none of malignance and worsening or recurrence of scars was observed.

**Lessons:**

MPR technology combined with post-operative hypofractionated electron-beam radiation therapy is an effective method for patients with multiple keloids distributed widely on the body with minimal complications, especially for patients with widely distributed keloids.

## 1 Introduction

Keloids are essentially products of abnormal wound healing resulting from an imbalance between the deposition and degradation of extracellular matrix, and often appear after surgery, trauma, infections, or burns.^[1]^ Unlike hypertrophic scars, keloids are characterized by the overproduction and deposition of type I and III collagen around numerous myofibroblasts, as seen histologically. In addition, keloids appear as fibrous nodules which always extend beyond the initial wound margin without regression over time.^[2]^ People with darker skin, that is, of African-American, Asian, or Hispanic descent, are much more vulnerable to keloids compared to those with European descent.^[3]^ Keloids can not only be aesthetically displeasing but also accompanied by symptoms of pain, itching, functional disabling, and so on. Unfortunately, there is no consensus at present on the optimal therapy of keloids, making it a great challenge for doctors.^[4]^

Management of keloids involves both noninvasive and invasive measures such as corticosteroid injections, administration of 5-fluorouracil, bleomycin, and interferon, silicone sheeting, cryotherapy, laser and light-based therapy, surgery, and radiotherapy.^[5]^ Although corticosteroid is usually recommended as the first-line treatment, there is no standard optimal therapy for keloids.^[6]^ A combination of the above methods is often used to optimize the outcome. For severe keloids that respond poorly to lasers and injection treatments, surgical excision with adjuvant irradiation can not only completely remove the keloids, but also greatly reduce the recurrence.^[7]^ However, there are some clinical limitations for surgical excision. It is not a practical option for patients with small, widely distributed keloids since a single excision cannot remove all the scars, and surgical excision of keloids around movable joints may lead to functional disabilities.

Micro-plasma radio-frequency (MPR) technology has been demonstrated as an effective therapy on rhytides, photo-aged skin, scars, and post-burn hyperpigmentation with rare complications and shorter recovery time.^[8–16]^ Since 2013, we have been developing a novel treatment for keloids, which combines MPR with hypo-fractionated electron-beam radiation therapy. The purpose of the present study is to investigate the clinical outcome of the novel treatment for keloids.

## 2 Methods

Twenty-two Asian patients (16 males and 6 females with ages 19–46 years and mean age 28.14 ± 7.31 years) were enrolled in our study from February 2013 to December 2016. All of patients with keloids were treated by MPR technology combined with postoperative hypofractionated electron-beam radiation therapy. Patient demographics, procedural details, and follow-up were collected. This study was approved by the institutional review board (IRB) of our Hospital (IRB number S-K197).

Patients were included if they had been suffering from keloids for at least 6 months, and were not responding to nonsurgical treatments. Nursing or pregnant women, patients with infections, skin burst or other skin diseases around the keloids, and patients who had undergone previous ablative skin treatment, had used isotretinoin within 6 months, and contraindicated for postoperative radiotherapy (age <12 years, radiosensitive locations) were excluded from this study.

The affected areas were first cleaned with suds and then anesthetized with 0.5% lidocaine (Yimin Pharmaceutical Co, Ltd, Beijing, China). A single MPR pulse was given with an MPR device (Alma Lasers, Israel). A rolled tip was passed over the scars 3 to 5 times at energy settings of 80 to 100 watts until the scar tissue was smooth and a reticular crust could be seen. The operated area was coated with 0.5% erythromycin ointment (Shuangji Pharmaceutical Co, Ltd, Beijing, China) and treated with an ice pack for 30 minutes to ease the pain and inflammation. Immediately after the treatment, the patients were asked to rate their pain level on a visual analog scale (VAS, scores 0–10).

The first radiotherapy was initiated within 24 hours after the radio-frequency treatment and the second was performed 1 week later with a cumulative dose of 18 Gy (9 Gy × 2). The electron beam was administered at 6 MeV, and the margin of the radiation field was 1 cm around the treated area. After each radiotherapy, the operative area was coated with erythromycin ointment. The patients were instructed to using sun cream (sun protection factor ≥30) till the time the crust naturally fell off to prevent photodamage and hyperpigmentation, and silicone gel can be used to prevent the scar formation twice a day for about 1 year. Fellow-up visits were arranged on 1 and 3 days, 1 week and at least 12 months after the treatment.

Digital photographs were taken before and 12 to 18 months after the second radiotherapy, and 2 expert plastic surgeons to assess the treatment effect. The pre- and post-treatment lesions were evaluated as per the vancouver scar scales (VSS)^∗^ (vancouver scar scales pre-treatment [VSS_pre_] and vancouver scar scales post-treatment [VSS_post_], respectively). The treatment efficacy was defined as the rate of decrease in VSS, that is, (VSS_pre_ − VSS_post_)/VSS_pre_, and graded as excellent (VSS decrease ≥90%), good (VSS decrease 60%–89%), fair (VSS decrease 30%–59%) or poor (VSS decrease ≤30%). After the first and third day of radio-frequency treatment, the patients were asked to rate their pain level with the VAS (0–10). At the initial follow-up, the patients were asked to grade their satisfaction using the following standard: extremely satisfied (score 3), satisfied (score 2), approximately satisfied (score 1), unsatisfied (score 0). Complications, including infections, erythema, worsening or recurrence of scars, hyperpigmentation or hypopigmentation and the time of epithelialization, were recorded for each case.

^∗^The VSS is a kind of classic evaluation scale for scar. VSS-total scores vascularity, pigmentation and height from 0 to 3 and pliability from 0 to 5. A 0 score resembles normal skin, while maximum scores indicate worst possible.

The VSS_pre_ and VSS_post_ scores were compared with paired *t* test, and the ΔVSS (VSS_pre_ − VSS_post_) were compared with unpaired *t* test. A 2-tailed *P*-value < .05 was considered statistically significant. All statistical analyses were conducted using SPSS 24.0.

## 3 Results

### 3.1 Demographic clinical characteristics

The average age of the patient cohort was 28.14 ± 7.31 years (range, 19–46 years). Etiologically, the cohort included 9 cases of acne, 4 of burn, and 9 of trauma. Lesions were located on the face and neck (14 cases), trunk (6 case), and limbs (2 cases). The average follow-up was 14.00 ± 2.02 months (range, 12–18 months). The detailed demographic data are shown in Table 1.

**Table 1:**
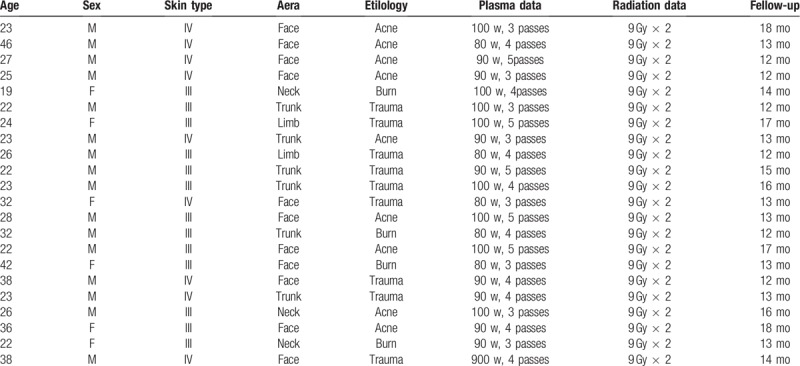
Patients’ demographic and operative data.

### 3.2 VSS and VAS scores

**Figure 1:**
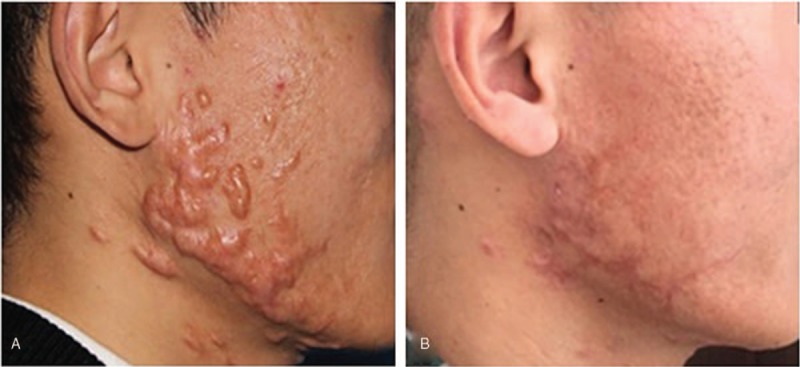
A 28 yr old man with acne scars on the right/left face. (A) Before treatment. (B) 13 mo after treatment of micro-plasma radio-frequency technology (100 w, 5 passes) combined with twice hypofractionated electron-beam radiation therapy (total dose of 18 Gy in 2 fractions).

**Figure 2:**
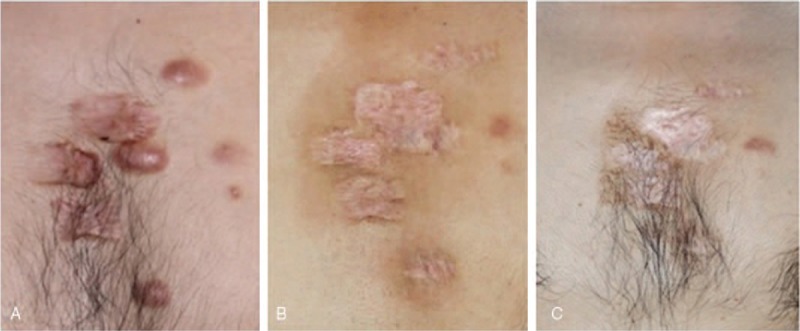
A 22 yr old man, trauma scars on chest. (A) Before treatment. (B) 4 mo after treatment, (C) 12 mo after treatment of micro-plasma radio-frequency technology (100 w, 3 passes) combined with hypofractionated electron-beam radiation therapy (9 Gy × 2).

**Figure 3:**
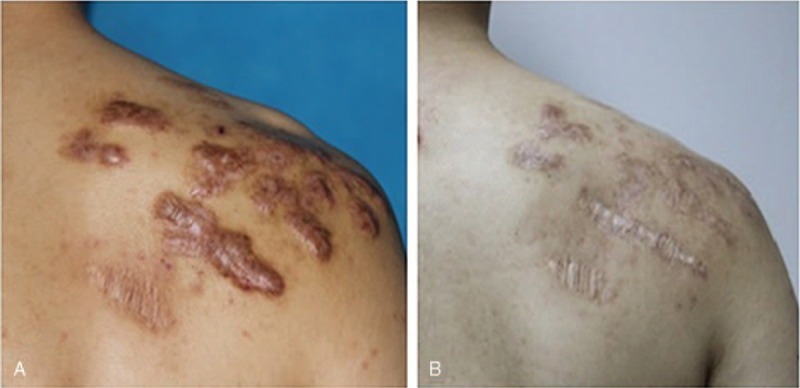
A 23 yr old man, acne scars on shoulder. (A) Before treatment. (B) 16 mo after treatment of micro-plasma radio-frequency technology (100 w, 4 passes) combined with hypofractionated electron-beam radiation therapy (9 Gy × 2).

**Figure 4:**
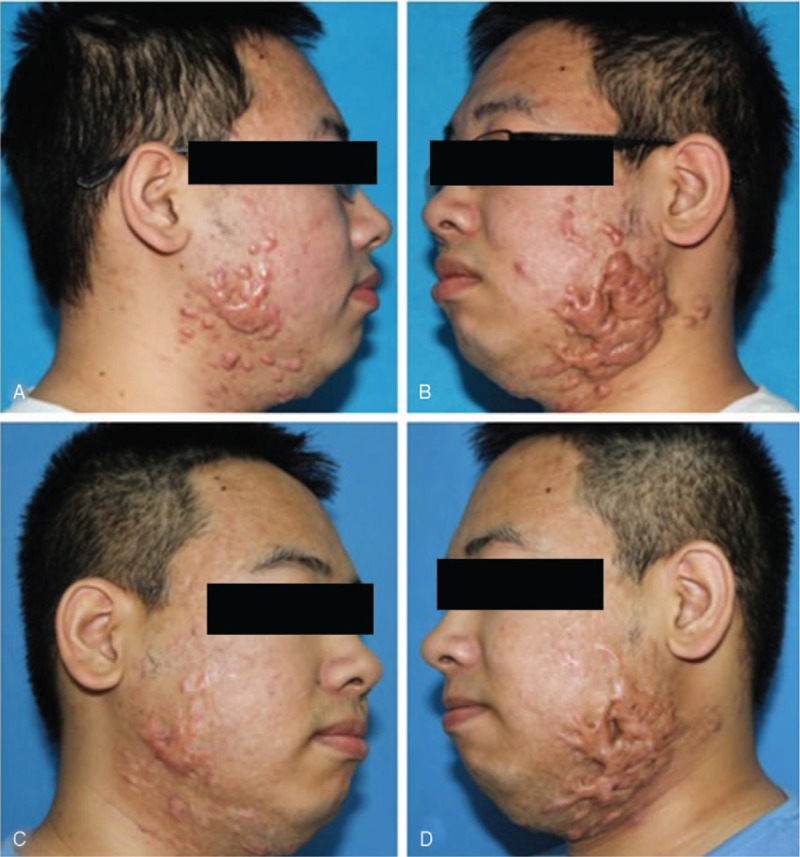
A 22 years old man, acne scars on face. (A and B) Before treatment. (C and D) 17 mo after treatment of micro-plasma radio-frequency technology (100 w, 5 passes) combined with hypofractionated electron-beam radiation therapy (9 Gy × 2).

The VSS scores decreased significantly 12 to 18 months after treatment (mean VSS_post_ = 5.77 ± 0.32, N = 22) compared to the pre-treatment (mean VSS_pre_ = 10.59 ± 0.30, N = 22) scores (*P* ≤ .001). And the ΔVSS of the patients with keloids on face and neck was 5.38 ± 0.69, while the ΔVSS of the patients with keloids on trunk and limbs was 5.70 ± 0.68 (*P* = .756). According to the rate of decrease in VSS, the improvement of scars was “good” in 16 patients (72.7%) and “fair” in 6 patients (27.3%); no “excellent” or “poor” recovery was seen. Four representative patients are shown in Figures 1 to 4. According to the patient satisfaction ratings, 21 patients were satisfied with their treatment, and 77.3% were extremely satisfied. Only 1 patient was dissatisfied due to the prolonged wound healing and pain. The pain was significant alleviated 3 days (1.27 ± 0.71) after the treatment compared to 1 day post-treatment (6.41 ± 1.41) (*P* ≤ .01). Seven days later, almost no patients complained of pain (mean VAS of 0.18).

### 3.3 Postoperative re-epithelialization time and complications

The average duration of re-epithelialization was 15.64 ± 3.54 days (range, 13–32 days). Only 1 patient had a wound-healing problem with prolonged re-epithelialization of 32 days. Apart from this case, no infection or prolonged wound healing was seen in any patient. Mild to moderate hyperpigmentation was observed in 3 patients 1 or 2 months post-treatment but they all recovered spontaneously within 6 months without any medical intervention. During the follow-up period, no malignant transformation or recurrence was observed (Table 2).

**Table 2:**
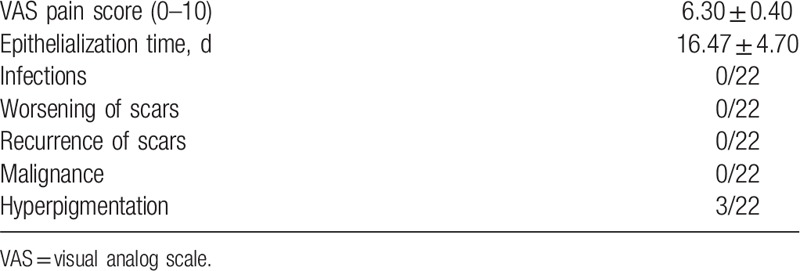
Complications.

## 4 Discussion

Keloids are caused by the deposition of disorganized collagen fibers during the process of abnormal wound healing. Owing to unknown etiology and high recurrence rate, keloids have been a great challenge to treat for both dermatologists and plastic surgeons. For better outcome, different methods are often used in combination, such as surgical excision with intralesional injection of corticosteroid, 5-fluorouracil, bleomycin and interferon, and surgical excision with adjuvant irradiation.^[5]^ For severe keloids that respond poorly to lasers and injection treatments, surgical excision with adjuvant irradiation can not only completely remove the keloids, but also greatly reduce the recurrence rate from >50% to 10.5%.^[7]^ That is mainly due to the anti-proliferative effect of radiotherapy on the fibroblasts which also negatively impacts neo-collagenesis.^[17]^ New developments in keloid radiotherapy focus on limited fractions and high radiation dose per fraction, and the hypo-fractionated electron-beam radiation has been demonstrated to be highly effective in reducing keloids recurrence rate with minimal complications.^[18,19]^ Compared to photon irradiation or kilo-voltage X-rays, hypo-fractionated electron-beam radiation can provide better protection to the underlying normal tissue and radiosensitive regions.^[20]^ A retrospective study of 568 cases from our hospital with 834 keloids was conducted by Shen et al^[19]^ who reported that postoperative (within 48 hours) hypo-fractionated electron-beam radiation (18 Gy in 2 fractions with a 1-week interval) can decrease the recurrence rate to 11.75%.

**Figure 5:**
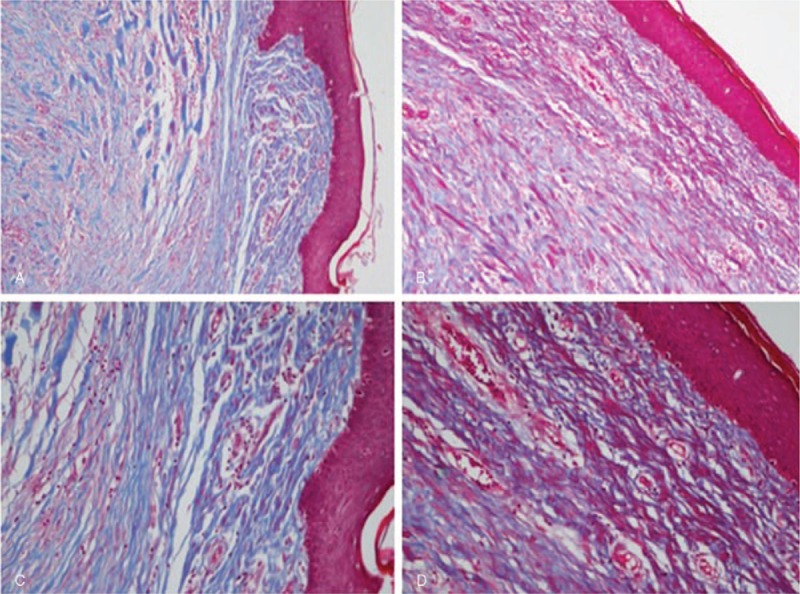
The histological examination showed over-proliferation of disorganized dermal collagen before treatment. By contrast, collagen remodeling and increased amount of new collagen deposition in the upper dermis were seen 3 mo after treatment. Biopsies stained by Masson dyeing: (A) immediately after treatment (100× magnification). (B) 3 mo after treatment (100× magnification). (C) Immediately after treatment (200× magnification). (D) 3 mo after treatment (200× magnification).

MPR technology is an emerging nonlaser, minimally ablative fractional treatment, which uses the manageable ultrahigh radiofrequency energy to ionize nitrogen into plasma, the fourth state of matter. The energy is applied onto the skin surface in the form of heat accompanied with electron capture, causing a deep thermal effect as the plasma is discharged from the distal end of a hand-held instrument without direct contact with skin. The thermal energy removes benign skin lesions and activates skin regeneration, which is histologically characterized by mild epidermal necrosis, continuous dermal neo-collagenesis, and collagen remodeling. And the Masson staining results of 1 of our patient's biopsy also demonstrated the collagen remodeling and increased amount of new collagen deposition 3 months after the treatment (Fig. 5). Unlike ablative lasers, MPR is nonchromophore dependent and the necrotic epidermis is not vaporized but retained as the natural “dressing,” which can accelerate wound healing with less side effects and shorter recovery time.^[8]^ Recent studies show that MPR is an effective therapy for moderate to deep rhytides, photo-aged skin, acne scars, mesh skin graft scars, traumatic scars, facial post-burn hyperpigmentation with rare complications, and shorter recovery time.^[8–16]^ Kono et al^[15]^ reported that the plasma treatment was a safe and effective method to treat traumatic scars with the mean re-epithelization time of 7.3 days, but did not recommended it for deep scars, such as abdominal surgery scars.

This is the first report on combining MPR technology with hypo-fractionated electron-beam radiation for keloid treatment. The volume of keloids reduced significantly in most patients, with significant improvement in texture (VSS_pre_ 10.59 ± 0.30 vs VSS_post_ 5.77 ± 0.32). The treatment effect of keloid is related to the pathogenic sites, and Ogawa et al concluded that the recurrence rate of keloids in high-tension sites (such as the chest wall, shoulder, and supraclavicular) was much higher than keloids in low tension sites (such as the earlobes, neck, and upper extremities).^[21]^ However, the results of our study shows that the difference between the ΔVSS of the patients with keloids on face and neck and the ΔVSS of the patients with keloids on trunk and limbs had no statistical significance (*P* > .05). One of the reasons may be that MPR is used in our treatment to make keloids tissue flat with the surrounding normal skin, while the wound after surgical excision of keloids needs to be sewed, which increases the local tension.

As mentioned above, surgery combined with local radiotherapy is an effective technique for refractory keloids. However, faced with the patients with multiple keloids on the special sites, such as chest, shoulder, and lower jaw as shown in Figures 1–4, excising the keloids at 1 time can result in excessive local tension and the inability to close the wound directly, which may increase the recurrence rate. Therefore, compared with traditional surgical resection, the most obvious advantage of MPR is that there is no increase in local tension around the treatment area, and we can remove multiple scattered keloids by just 1 treatment. After treated by MPR in our study, the keloids tissue was flat with the surrounding normal skin, and the accompanying symptoms of pain, itching were alleviated. Based on our results, 21 of 22 patients were satisfied with their treatment, and 17 patients were extremely satisfied.

In addition, the scar-related pain and itching was alleviated. All patients received “good” (16/22) or “fair” (6/22) results, and most patients were basically content with the treatment. As shown in the digital photographs, improvement in keloid texture was greater in the peripheral regions compared to the core areas, and the reason may be that the area surrounding the keloids were closer to normal skin texture. The discomfort and pain were most severe on the first day after treatment (VAS = 6.41 ± 1.41), which was a main cause of patients’ dissatisfaction. Although we had anesthetized the operative area with lidocaine hydrochloride, better strategies are needed for perioperative pain monitoring. The mean re-epithelization time was 15.64 ± 3.54 days, about 9 days longer than what Kono et al^[15]^ had reported. It is probably because postoperative radiotherapy repressed the over-proliferation of fibroblasts as well as neo-collagenesis and neovascularization, due to the higher energy settings in our study.

There are some limitations of the study. We observed lower treatment efficacy with thick keloids, along with longer re-epithelization time, higher risk of infection and hyperpigmentation, and poor anesthetic penetration. All of the above can decrease patients’ satisfaction. As shown in Figure 4, keloids on the left side were too thick to achieve a smooth surface post-treatment compared to the right side. In addition, this combination treatment is not recommended for patients who are contraindicated for radiotherapy (eg, younger than 12 years), and on radiosensitive locations like breasts and thyroid. Furthermore, a study with a control-group is still needed to prove the efficacy and safety of the novel therapy and compare the therapeutic response of keloids with different thickness.

## 5 Conclusions

To sum up, MPR technology combined with hypo-fractionated electron-beam radiation therapy is safe and clinically efficient for severe keloids that respond poorly to lasers and injection treatments. We recommend the combination of MPR and hypo-fractionated electron-beam radiation for the patients with multiple keloids distributed widely on the body which is not suitable for surgical resection. MPR treatment can make the keloids tissue almost flat with the surrounding normal skin, and hypofractionated electron-beam radiation can reduce the recurrence rate. Nevertheless, it is important to remember that surgical excision is still the first choice for sever keloids, and the novel therapy is an excellent alternative. Further studies are needed to investigate the effect of MPR technology in combination with other adjuvant therapies like irradiation and intralesional therapies.

## Author contributions

**Conceptualization:** Zhifei Liu, Nanze Yu.

**Data curation:** Zhifei Liu, Lin Zhu, Wenyun Ting, Xiaojun Wang.

**Formal analysis:** Zhifei Liu.

**Investigation:** Wenchao Zhang, Zhifei Liu, Ang Zeng, Wenyun Ting.

**Methodology:** Wenchao Zhang, Lin Zhu, Nanze Yu, Guangpeng Xia.

**Project administration:** Wenchao Zhang, Zhifei Liu, Nanze Yu.

**Resources:** Wenchao Zhang, Wenyun Ting, Nanze Yu.

**Software:** Lin Zhu.

**Supervision:** Zhifei Liu, Ang Zeng, Xiaojun Wang.

**Validation:** Lin Zhu, Guangpeng Xia.

**Visualization:** Zhifei Liu, Ang Zeng, Guangpeng Xia.

**Writing – original draft:** Wenchao Zhang, Guangpeng Xia.

**Writing – review and editing:** Wenchao Zhang, Ang Zeng, Xiaojun Wang.

## References

[1] Tredget EE Pathophysiology and treatment of fibroproliferative disorders following thermal injury. Ann N Y Acad Sci 1999;888:165–82. 10842632 10.1111/j.1749-6632.1999.tb07955.x

[2] Gauglitz GG Korting HC Pavicic T Hypertrophic scarring and keloids: pathomechanisms and current and emerging treatment strategies. Mol Med 2011;17:113–25. 20927486 10.2119/molmed.2009.00153 PMC3022978

[3] Jones ME Hardy C Ridgway J Management: a retrospective case review on a new approach using surgical excision, platelet-rich plasma, and in-office superficial photon X-ray radiation therapy. Adv Skin Wound Care 2016;29:303–7. 27300360 10.1097/01.ASW.0000482993.64811.74 PMC4915758

[4] Del Toro D Dedhia R Tollefson TT Advances in scar management: prevention and management of hypertrophic scars and keloids. Curr Opin Otolaryngol Head Neck Surg 2016;24:322–9. 27163611 10.1097/MOO.0000000000000268

[5] Arno AI Gauglitz GG Barret JP Up-to-date approach to manage keloids and hypertrophic scars: a useful guide. Burns 2014;40:1255–66. 24767715 10.1016/j.burns.2014.02.011 PMC4186912

[6] Gold MH McGuire M Mustoe TA Updated international clinical recommendations on scar management: part 2 – algorithms for scar prevention and treatment. Dermatol Surg 2014;40:825–31. 25068544 10.1111/dsu.0000000000000050

[7] van Leeuwen MC Stokmans SC Bulstra AE Surgical excision with adjuvant irradiation for treatment of keloid scars: a systematic review. Plast Reconstr Surg Glob Open 2015;3:e440. 26301129 10.1097/GOX.0000000000000357 PMC4527614

[8] Foster KW Moy RL Fincher EF Advances in plasma skin regeneration. J Cosmet Dermatol 2008;7:169–79. 18789051 10.1111/j.1473-2165.2008.00385.x

[9] Bentkover SH Plasma skin resurfacing: personal experience and long-term results. Facial Plast Surg Clin North Am 2012;20:145–62. 22537783 10.1016/j.fsc.2012.02.010

[10] Kilmer S Semchyshyn N Shah G A pilot study on the use of a plasma skin regeneration device (Portrait PSR3) in full facial rejuvenation procedures. Lasers Med Sci 2007;22:101–9. 17342383 10.1007/s10103-006-0431-9

[11] Potter MJ Harrison R Ramsden A Facial acne and fine lines: transforming patient outcomes with plasma skin regeneration. Ann Plast Surg 2007;58:608–13. 17522481 10.1097/01.sap.0000252481.84134.fe

[12] Alster TS Konda S Plasma skin resurfacing for regeneration of neck, chest, and hands: investigation of a novel device. Dermatol Surg 2007;33:1315–21. 17958582 10.1111/j.1524-4725.2007.33282.x

[13] Higashimori T Kono T Sakurai H Treatment of mesh skin grafted scars using a plasma skin regeneration system. Plast Surg Int 2010;2010:874348. 22567232 10.1155/2010/874348 PMC3335560

[14] Gonzalez MJ Sturgill WH Ross EV Treatment of acne scars using the plasma skin regeneration (PSR) system. Lasers Surg Med 2008;40:124–7. 18306162 10.1002/lsm.20617

[15] Kono T Groff WF Sakurai H Treatment of traumatic scars using plasma skin regeneration (PSR) system. Lasers Surg Med 2009;41:128–30. 19226574 10.1002/lsm.20723

[16] Wang LZ Ding JP Yang MY Treatment of facial post-burn hyperpigmentation using micro-plasma radiofrequency technology. Lasers Med Sci 2015;30:241–5. 25209007 10.1007/s10103-014-1649-6

[17] Ogawa R Mitsuhashi K Hyakusoku H Postoperative electron-beam irradiation therapy for keloids and hypertrophic scars: retrospective study of 147 cases followed for more than 18 months. Plast Reconstr Surg 2003;111:547–53. 12560675 10.1097/01.PRS.0000040466.55214.35

[18] Duan Q Liu J Luo Z Postoperative brachytherapy and electron beam irradiation for keloids: a single institution retrospective analysis. Mol Clin Oncol 2015;3:550–4. 26137265 10.3892/mco.2015.498 PMC4471515

[19] Shen J Lian X Sun Y Hypofractionated electron-beam radiation therapy for keloids: retrospective study of 568 cases with 834 lesions. J Radiat Res 2015;56:811–7. 26224888 10.1093/jrr/rrv031 PMC4577000

[20] Bischof M Krempien R Debus J Postoperative electron beam radiotherapy for keloids: objective findings and patient satisfaction in self-assessment. Int J Dermatol 2007;46:971–5. 17822505 10.1111/j.1365-4632.2007.03326.x

[21] Ogawa R Miyashita T Hyakusoku H Postoperative radiation protocol for keloids and hypertrophic scars: statistical analysis of 370 sites followed for over 18 months. Ann Plast Surg 2007;59:688–91. 18046154 10.1097/SAP.0b013e3180423b32

